# Correlation Between Pancreatic Islet Uncoupling Protein-2 (UCP2) mRNA Concentration And Insulin Status in Rats

**DOI:** 10.1155/EDR.2000.185

**Published:** 2000

**Authors:** Nadim Kassis, Catherine Bernard, Aristide Pusterla, Louis Casteilla, Luc Pétnicaud, Denis Richard, Daniel Ricquier, Alain Ktorza

**Affiliations:** 1 Laboratoire de Physiopathologie de la Nutrition CNRS ESA 7059 Université Paris 7-Denis Diderot Paris France; 2 Département de Physlologe Unversité Laval Québec Canada; 3 CCNRS ESA 5018 Université Paul Sabatier Toulouse France; 4 CEREMOD CNRS UPR 9078 9, rue Jules Hetzel Meudon 92190 France

## Abstract

Hypothesizing that UCP2 may influence insulin secretion by modifying the ATP/ADP ratio within pancreatic islets, we have investigated the expression of intraislet UCP2 gene in rats showing insulin oversecretion (non-diabetic Zucker fa/fa obese rats, glucose-infused Wistar rats) or insulin undersecretion (fasting and mildly diabetic rats). We found that in Zucker fa/fa obese rats, hyperinsulinemia (1222 ± 98 pmol/1 *vs*. 128 ± 22 pmol/1 in lean Zucker rats) was accompanied by a significant increase in UCP2 mRNA levels. In rat submitted to a 5 day infusion with glucose, hyperinsulinemia (1126 ± 101 pmol/l *vs*. 215 ± 25 pmol/1 in Wistar control rats), coincided with an enhanced intraislet UCP2 gene expression, whereas a 8h or a 2 day-infusion did not induce significant changes in UCP2 mRNA expression. In rats made hypoinsulinemic and mildly diabetic by the injection of a low dose of streptozotocin, and in 4-day-fasting rats (plasma insulin 28 ± 5 pmol/1) UCP2 gene expression was sharply decreased. A 3-day-fast was ineffective. The data show the existence of a time-dependent correlation between islet mRNA UCP2 and insulin that may be interpreted as an adaptative response to prolonged insulin excess.


*Int. Jnl. Experimental Diab. Res.*, Vol. 1, pp. 185–193
Reprints available directly from the publisher
Photocopying permitted by license only

© 2000 OPA (Overseas Publishers Association) N.V.
Published by license under
the Harwood Academic Publishers imprint,
part of The Gordon and Breach Publishing Group.
Printed in Malaysia.

### Correlation Between Pancreatic Islet Uncoupling Protein-2 (UCP2) mRNA Concentration and Insulin Status in Rats

NADIM KASSIS[a], CATHERINE BERNARD[a], ARISTIDE PUSTERLA[b], LOUIS CASTEILLA[c], LUC PÉNICAUD[c], DENIS RICHARD[b], DANIEL RICQUIER[d] and ALAIN KTORZA[a,*]

[a]*Laboratoire de Physiopathologie de la Nutrition, CNRS ESA 7059, Université Paris 7-Denis Diderot, Paris, France;* [b]*Département de Physiologie, Université Laval, Québec, Canada;* [c]*CNRS ESA 5018, Université Paul Sabatier, Toulouse, France;* [d]*CEREMOD CNRS UPR 9078, 9, rue Jules Hetzel, Meudon, 92190-France*

*(Received in final form 8 March 2000)*

**Hypothesizing that UCP2 may influence insulin secretion by modifying the ATP/ADP ratio within pancreatic islets, we have investigated the expression of intraislet UCP2 gene in rats showing insulin oversecretion (non-diabetic Zucker fa/fa obese rats, glucose-infused Wistar rats) or insulin undersecretion (fasting and mildly diabetic rats). We found that in Zucker fa/fa obese rats, hyperinsulinemia (1222 ± 98 pmol/l *vs.* 128 ± 22 pmol/l in lean Zucker rats) was accompanied by a significant increase in UCP2 mRNA levels. In rat submitted to a 5 day infusion with glucose, hyperinsulinemia (1126 ± 101 pmol/l *vs.* 215 ± 25 pmol/l in Wistar control rats), coincided with an enhanced intraislet UCP2 gene expression, whereas a 8h or a 2 day-infusion did not induce significant changes in UCP2 mRNA expression. In rats made hypoinsulinemic and mildly diabetic by the injection of a low dose of streptozotocin, and in 4-day-fasting rats (plasma insulin 28 ± 5 pmol/l) UCP2 gene expression was sharply decreased. A 3-day-fast was ineffective. The data show the existence of a time-dependent correlation between islet mRNA UCP2 and insulin that may be interpreted as an adaptative response to prolonged insulin excess.**

*Keywords*: UCP2; Plasma insulin; Hyperinsulinemic rats; Hypoinsulinemic rats; Fasting; Glucose infusion

### INTRODUCTION

The uncoupling protein-1 (UCP1) mediates the proton leak in the mitochondria of the brown adipose tissue (BAT) and promotes uncoupling fuel oxidation from ADP phosphorylation.[1, 2] This process is involved in thermoregulation and the control of energy balance.[3] Until recently, the contribution of this pathway to the regulation of energy expenditure and ATP synthesis was considered as restricted to neonate mammalians and rodents because BAT is present only in small amounts in non-rodent adult mammalians including humans.[2]

The discovery of homologs of UCP *i.e.*, UCP2[4, 5] and UCP3,[6–8] and more recently of

*Corresponding author. Laboratoire de Physiopathologie de la Nutrition, CNRS ESA 7059, 2, Place Jussieu, Tour 23-33-1er tage, 75251 Paris cedex 05-France. Tel.: (33-1) 44 27 78 37, Fax: (33-1) 44 27 78 36, e-mail: ktorza@paris7.jussieu.fr

UCP4 [9] whose expression is not limited to BAT led to re-evaluate this point of view. These new isoforms present similar properties as UCP1 in terms of uncoupling capacity.

This situation arises the question of the specific role of UCPs in tissues or organs whose function is closely dependent on the ATP/ADP ratio. This is particularly true for insulin secretion by the endocrine ß-cell of the pancreas. The stimulation of insulin release by glucose is initiated by the closure of ATP dependent $K^+$ channels by the ATP originating from glucose metabolism. This results in membrane depolarization and then a massive influx of $Ca^{2+}$ through the opening of $Ca^{2+}$ channels that stimulates exocytosis of insulin vesicles.[10] Therefore, the ATP/ADP ratio in the ß-cell is of crucial importance in the secretory process.

Considering that UCP2 may influence insulin secretion by modifying the ATP/ADP ratio, we have investigated the *in vivo* expression of UCP2 gene in the endocrine pancreas under conditions of over activity (non-diabetic hyperinsulinemic Zucker fa/fa rats, glucose-infused Wistar rats) and underactivity (fasting and mildly diabetic rats) of the ß-cell. We concentrated on UCP2 because it has been detected in the pancreas [4, 11] contrarily to the other isoforms. This study was further stimulated by the fact that the UCP2 gene is localized to a region of rat chromosome 1 linked to glucose homeostasis in the Wistar rat.[12] This region also includes the gene for the sulphonylurea receptor,[13] involved in insulin secretion.[14]

### MATERIALS AND METHODS

### Animals

Three-month-old male Wistar rats were used. They were maintained at 24 ± 2°C in a room with a 12h fixed light-dark schedule. They had free access to water and standard laboratory chow pellets. For fasting experiments, Wistar rats were submitted to fasting during 4 days. Three-month-old genetically obese (fa/fa) non-diabetic male Zucker rats [15] were used as a model of spontaneous long-standing hyperinsulinemic rats. Lean (Fa/?) male Zucker rats were used as controls. Zucker rats were housed under the same conditions as Wistar rats.

Diabetes was induced in Wistar rats by a single i.v. injection through the saphenous vein of 35 mg/kg body weight streptozotocin (STZ, Sigma, St. Louis, Mo, USA) dissolved in a citrate buffer (0.1mol/l, pH4.5). Controls were i.v. injected with citrate buffer.

Wistar rats were submitted to saline (0.9% NaCl) or glucose infusions. Infusions were carried out with the long-term infusion technique under unrestrained conditions as previously described.[16] Hypertonic (30% wt/volume) glucose (Chaix et Du Marais, Paris, France) was infused at an initial rate of 50μl/min to produce hyperglycemia around 20mmol/l. Glycemia was maintained in the required ranges by adjusting the infusion flow rate throughout the infusion period. The infusion lasted either 8 h (hyperglycemic-hyperinsulinemic rats: HGHI 8 h rats), or 2 days (HGHI 2 days rats), or 5 days (HGHI 5 days rats). Rats in which glycemia did not stay around 20mmol/l were excluded.

### Insulin and Glucose Measurements

The STZ diabetic state was characterized by the measurement of basal plasma glucose concentration and an i.v. glucose tolerance test (IVGTT) performed two weeks after STZ injection. Glucose (0.8g/kg body weight) was injected in the postabsorptive state under pentobarbital anesthesia (4mg/100g body weight, i.p.-Sanofi Santé Animale, Libourne, France). Blood samples were collected sequentially before and after glucose injection. Insulin and glucose responses during IVGTT were calculated as the ΔI and the G. The ΔI is the incremental plasma insulin values integrated over a period of 30min after the injection of glucose. The ΔG is the corresponding increase in glucose concentration. The insulinogenic index ($\Delta I/\Delta G$) represents the ratio of these two parameters.

In another group of rats, basal plasma glucose and insulin concentrations were measured 2 days after STZ injection.

### Hyperglycemic and Hyperinsulinemic Wistar Rats

During 8 h or 2 days or 5 days infusions, plasma glucose and insulin concentrations were measured on blood collected by tail snipping, five times daily in glucose-infused rats and only twice daily in saline-infused rats where these parameters remained very stable.

### Zucker and Fasted Wistar Rats

Plasma glucose and insulin concentrations were measured on blood collected by tail snipping, in rats in the post-absorptive state.

*In situ* hybridization histochemistry was used to ascertain the presence of UCP2 mRNA within the pancreatic islets. The protocol used was adapted from the technique of Simmons *et al.*[17] as previously described.[18] A rat pancreas was dissected out following an intracadial perfusion with paraformaldehyde. The pancreas was thereafter embedded in parrafin and sliced using a rotory microtome. The pancreas sections, which were 5 μm thick, were mounted on slides and hybridized with a radioactive UCP2 cRNA probe. The specificity of the antisense riboprobe was strongly verified by the total absence of positive signal in a pancreas section hybridized with the sense probe.

### Northern Blot Analysis

Islets were isolated by collagenase digestion (Collagenase, Boehringer Mannheim, Mannheim, Germany). Islet total RNA were extracted using Chomczynski's method.[19] The A260/280 ratios and the 28S/18S RNA ratios were greater than 2. Northern blot analysis was performed using 20 μg of total RNA. Because about 30 μg of RNA were obtained per rat, one individual rat was used for each Northern blot point. After blotting, nylon membranes were successively hybridized with UCP2 and 18S probes, labelled with ($\alpha$32P)-dCTP using a Random primed DNA labeling kit (Boehringer Mannheim Biochemica, Germany). The cDNA probe of UCP2 comprised the complete coding sequence (935 bp) of the mouse UCP2 (Genbank accession number U69135). Quantification was performed by densitometry, using an image analyser (Biocom, Les Ulis, France). The densitometric values of the autoradiograms were corrected for the intensities of the 18S RNA bands.

### Analytical Methods

Blood glucose was determined using a glucose analyzer (Beckman Inc, Fullerton, CA, USA). Insulin was measured by a radioimmunoassay kit (Sorin, Roma, Italy). The lower limit of the assay was 19.5 pmol/l with 6% coefficient of variation within and between the assay.

### Data Presentation and Statistical Methods

Data are presented as means ± SEM. The significance of the differences between means was evaluated by a one or two-way analysis of variance (ANOVA). A $p$ value $< 0.05$ was considered significant.

### RESULTS

Because all parameters were very similar in the different control groups of rats (saline-infused, non-fasting Wistar rats, citrate injected rats), we chose to represent the results concerning saline-infused rats as representative of the different Wistar control groups.

### Plasma Glucose and Insulin Concentrations

### *Lean and Obese Zucker Rats*

In lean and obese Zucker rats, plasma glucose concentration was similar to that of control Wistar rats (Fig. 1). Plasma insulin was 10-fold higher in obese Zucker rats compared to lean Zucker rats (Fig. 1).

### *Glucose-infused Rats*

Sustained and steady hyperglycemia of about 20mmol/l was produced in rats infused with glucose during 8h, or 2 days or 5 days. As a result, insulinemia was dramatically increased (Fig. 1). In both hyperglycemic-hyperinsulinemic groups, the glucose and insulin plateaus were reached after 4h of infusion with glucose (data not shown).

### *STZ Rats*

Two days after STZ injection, plasma glucose and insulin concentrations were, as compared to controls, significantly increased and decreased, respectively (Fig. 3).

Fifteen days after STZ injection, STZ rats were hyperglycemic and hypoinsulinemic compared to control Wistar rats (Figs. 2 and 3). Moreover, glucose tolerance and glucose-induced insulin secretion were impaired as reflected in the $\Delta G$ and the $\Delta I/\Delta G$, respectively (Fig. 2). Particularly, the insulin response was $\sim$3.5 fold lower in STZ rats than in control Wistar rats.

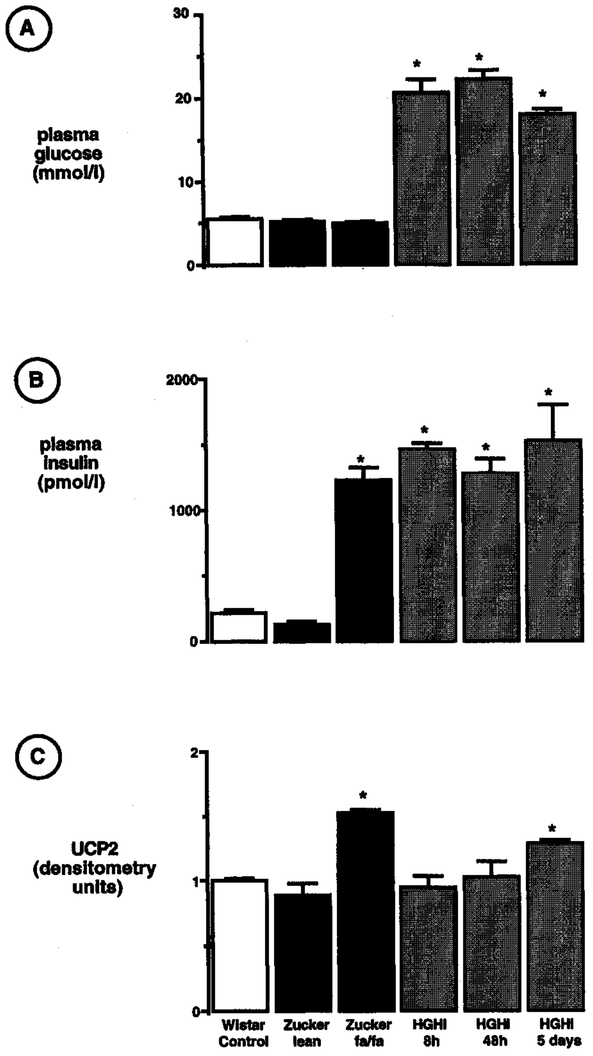

FIGURE 1 A and B: Plasma glucose (A) and insulin (B) in control rats and the different groups of hyperinsulinemic rats. C: Northern blot analysis and quantification of the relative levels of UCP2 mRNA in pancreatic islets in normo- and hyperinsulinemic rats. The densitometry of autoradiogram was corrected for 18S RNA. Data are means ± SEM of 4–7 rats. ⋆ $p < 0.05$ compared with Wistar control or lean Zucker rats. HGHI: hyperglycemic and hyperinsulinemic rats.

### *Fasting Wistar Rats*

A 3 day fast resulted in a slight decrease in plasma glucose concentration which was accompanied by a much more important fall in plasma insulin level, which was $\sim$ 7-fold lower than in control Wistar rats. Both parameters did not evolve significantly between 3 and 4-day fasting (Fig. 3).

### *In Situ* Histochemistry and Histology

The presence of UCP2 within the pancreatic islets was clearly demonstrated by *in situ*

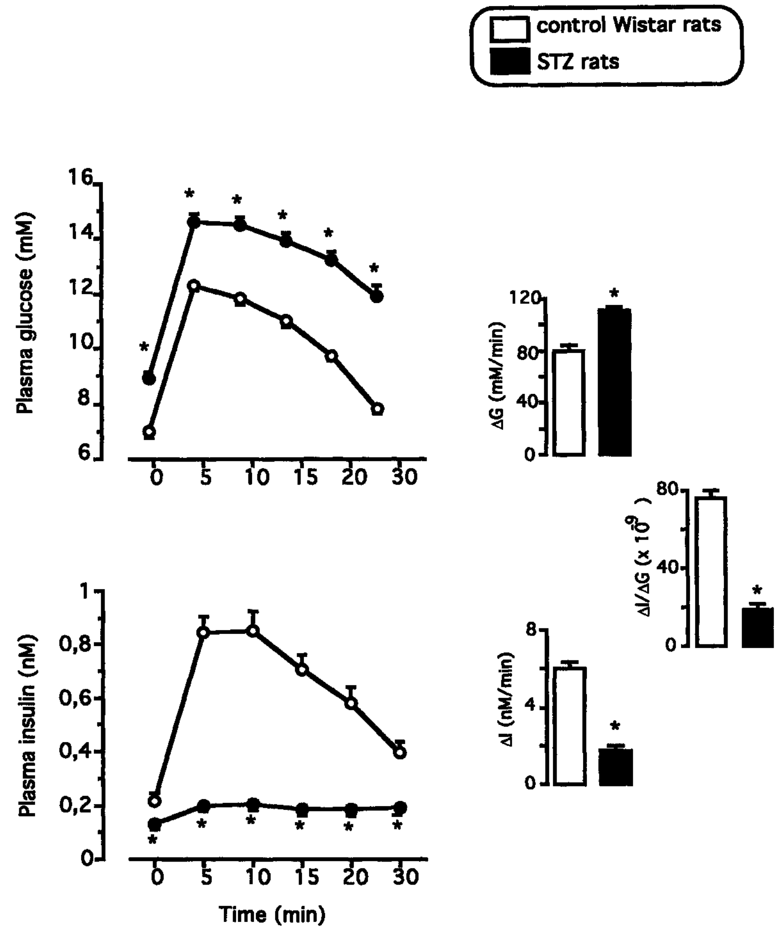

FIGURE 2 Time course of plasma glucose and insulin concentrations during an intravenous glucose tolerance test (IVGTT) performed in Wistar control and diabetic (STZ) rats two weeks after streptozotocin or citrate injection. Values are mean ± SEM. Number of rats was 7 for Wistar control and 5 for STZ rats. ⋆ $p < 0.05$ compared with Wistar control.

histochemistry (Fig. 4). The silver grains were distributed over the islets suggesting that UCP2 was expressed in more than one cell type. The presence of silver grains in areas stained in blue/purple following aldehyde-fuchsin staining strongly suggested the presence of UCP2 in ß-cells.

### UCP2 mRNA Levels

In Zucker fa/fa rats UCP2 mRNA levels were significantly increased compared to Zucker lean and Wistar control rats (Fig. 1). Neither a 8h nor a 2-day infusion with glucose did not result in any significant change in UCP2 gene expression, whereas rats infused with glucose during 5 days showed higher UCP2 mRNA levels than controls (Fig. 1). In STZ rats studied 15 days after the injection of the drug, UCP2 mRNA levels were clearly decreased compared to Wistar non diabetic rats (Fig. 2). When the rats were studied 2 days after the injection no change in islet UCP2 gene expression could be observed. Islet UCP2 gene expression was also markedly lowered after a 4-day fast but a 3-day fast failed to alter this parameter (Fig. 2).

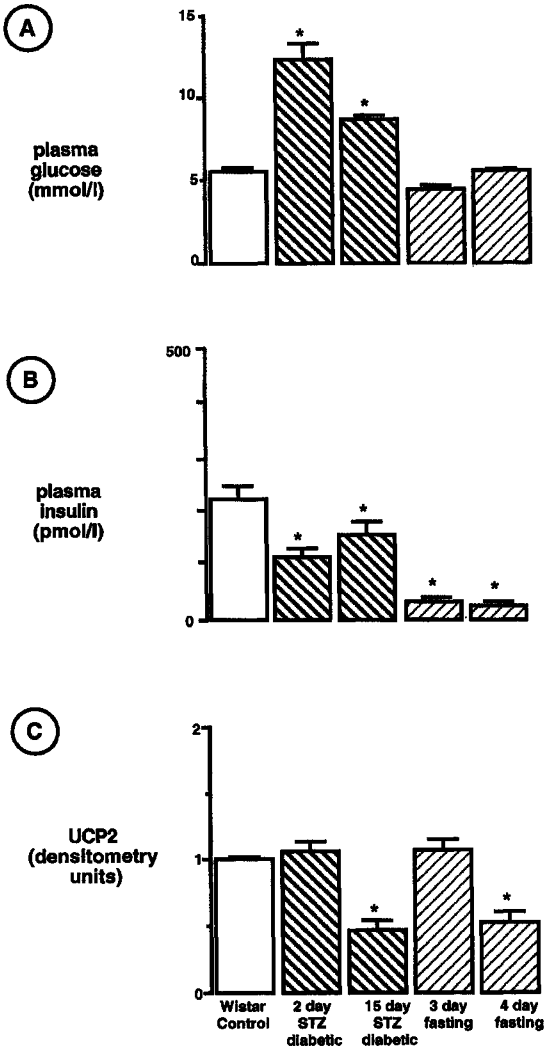

FIGURE 3 A and B: Plasma glucose (A) and insulin (B) in control and the different groups of hypoinsulinemic rats. The value for 15 day-STZ rats is the value at time point 0 of the intravenous glucose tolerance test. C: Northern blot analysis and quantification of the relative levels of UCP2 mRNA in pancreatic islets in normo- and hyporinsulinemic rats. The densitometry of autoradiogram was corrected for 18S RNA. Data are means ± SEM of 4–7 rats. ⋆ $p < 0.05$ significantly different from controls Wistar rats. STZ rats: Hypoinsulinemic and mildly diabetic rats.

### DISCUSSION

Both *in situ* hybridization and Northern blot analysis clearly indicate the expression of UCP2 in rat pancreatic islets in agreement with studies from other groups showing UCP2 gene expression in the whole pancreas [4] and pancreatic islets [11,20] in humans and rodents. The data obtained in our study provide evidence for a correlation *in vivo* between UCP2 gene expression in pancreatic islets and the insulin status in Zucker and Wistar rats.

UCP2 mRNA levels were clearly increased in non-diabetic fa/fa obese Zucker rats which are highly hyperinsulinemic and normoglycemic. Hyperinsulinemia associated with insulin resistance has been long recognized as one of the main characteristics of these rats.[15] Oversecretion of insulin in response to many secretagogues is present very early, before weaning *i.e.,* when obesity is not yet detectable.[21]

To confirm and precise the relationship between UCP2 mRNA and insulin release *in vivo,* we infused Wistar rats with glucose to provoke hyperinsulinemia and the infusion rate was adjusted to reach plasma insulin levels similar to those of obese fa/fa Zucker rats. When glucose infusion and consequently hyperinsulinemia lasted 5 days, an increase in UCP2 mRNA was observed. On the contrary, the increase in plasma insulin concentration resulting from a 8h or even a 2 day infusion with glucose, was not accompanied by significant changes in islet UCP2 mRNA. The fact that insulin oversecretion precedes the increase in islet UCP2 mRNA suggests that the increase in UCP2 gene expression could be a consequence of insulin oversecretion but cannot be an inducer of increased pancreatic islet function. Moreover, combined with the fact that hyperinsulinemia was established for a very long time in 3 month-old fa/fa obese Zucker rats used in our study, this suggests that, apart plasma insulin level, the duration of insulin oversecretion is of cucial importance in islet UCP2 gene expression.

If the level of UCP2 mRNA in pancreatic islets is correlated with islet function it could logically be expected that pancreatic islet underfunction is accompanied by UCP2 mRNA underexpression. To assess this point we used two models.

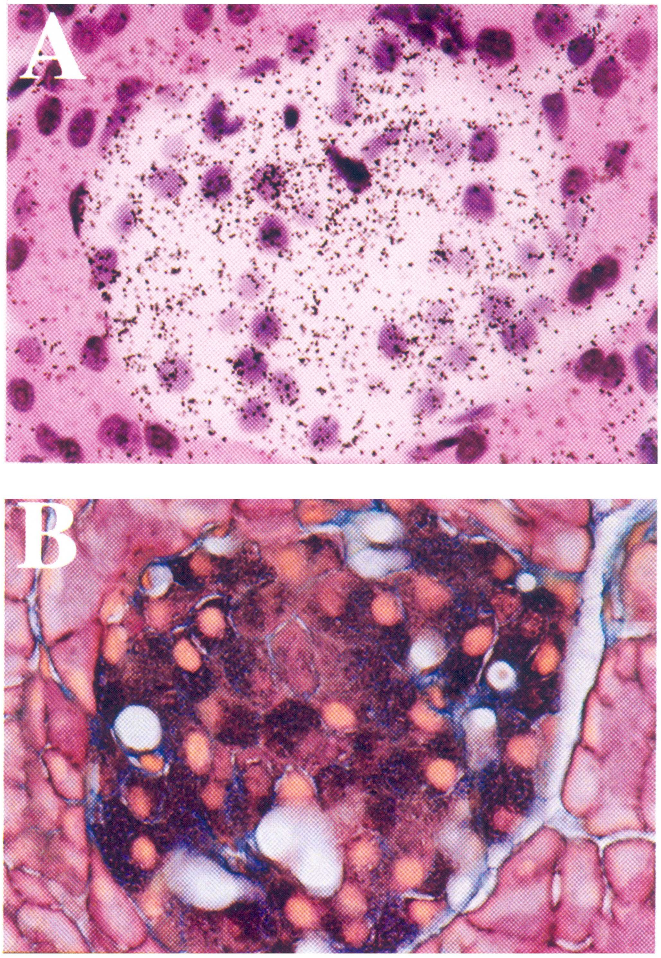

FIGURE 4 Panel A. Pancreatic islet hybridized with an antisense riboprobe complementary to the rat UCP2 mRNA. UCP2 mRNA is revealed by silver grains on a hematoxylin and eosin stained section. Panel B. Fuchsin staining of a pancreatic islet from a tissue section consecutive to the section seen in A. The ß-cells granules are stained in blue/purple whereas the $\alpha$-cells granules are stained in orange. Magnification 100 $x$. (See Color Plate I).

In the first model, Wistar rats were rendered mildly diabetic by the injection of a low dose of streptozotocin. Although basal insulin was only slightly decreased in STZ rats compared to controls, the very low insulin response to glucose loading strongly suggests that postprandial insulin secretion was greatly altered and therefore pancreatic islet activity was, as a whole, much lower than normal. As a result, UCP2 mRNA was sharply decreased. We found no change in islet UCP2 mRNA concentration 2 days after STZ injection, thus indicating that STZ *per se* could not influence UCP2 mRNA expression. A recent study by Kageyama *et al.*, showed an increased level of UCP2 mRNA in muscles of rats with strepozotocin-induced diabetes.[24] It is difficult to compare the data of this study and those of our own study because of the difference in the severity of experimental diabetes. However, this suggests that the regulation of UCP2 gene expression is tissue-specific. A second way to lower pancreatic islet function was to submit rats to fasting. As expected, fasting resulted in a sharp decrease in plasma insulin concentration whatever its duration but UCP2 mRNA was down-regulated only in 4-day fasting rats. This is a further argument in favor of the time-dependency of the effect of pancreatic islet activity on UCP2 gene expression. This time-dependency suggests that the influence of ß-cell function on islet UCP2 gene expression may require undirect mechanisms.

In rats, differential effects of fasting on UCP2 mRNA could be observed according to the tissue studied. UCP2 was up-regulated by a 48h fasting or food deprivation in skeletal muscle but neither in heart nor in brown adipose tissue (BAT) where it was unchanged.[27] Long-term food deprivation decreased UCP2 mRNA in interscapular BAT.[28] Taken together with the data from the present study, this tissue specificity gene expression argues for a specific regulation and role of UCP2 in pancreatic islets.

The main conclusion of this study is the existence of a quantitative and apparently time-dependent correlation between islet mRNA UCP2 and islet function. If a specific role is ascribed to islet UCP2, this correlation may be interpreted as an adaptative response to prolonged insulin excess. In situations of pancreatic islet overfunction, such as in obesity, UCP2 expression would be increased and in turn that would limit insulin secretion by lowering ATP concentration within the ß-cell. In a recent study, Wang *et al.*[29] induced UCP2 gene overexpression in islets of ZDF rats. They observed that this overexpression resulted in a decrease in ATP content within the islet and a concomitant restoration of glucose-induced insulin secretion. According to the authors conclusion, the data could be intrepreted in the following way: in ZDF rats, intraislet high ATP levels would permanently close $K^+$-ATP channels and finally will be responsible for the chronic basal insulin secretion observed in this model.[30] Consequently, the ß-cell would be insensitive to a further stimulation by glucose. The restoration of the insulin response to glucose by high UCP2 expression could therefore be explained by its lowering effect on ATP concentration which will abolish the permanent closure of $K^+$-ATP channels and the resulting insulin oversecretion. This cascade of events will allow the ß-cell to be normally sensitive to glucose. Another recent study showing a clear inhibition of the insulin response to glucose in rat islets overexpressing UCP2 also agrees with our hypothesis.[31] Altogether the data from both studies fit to our own data since they may ascribe to intraislet UCP2 a role of regulator of insulin secretion and reinforce the notion of a relationship between intraislet UCP2 expression and insulin secretion. Further experiments are required to explore the intracellular signal(s) linking UCP2 gene expression and insulin secretion.

### *References*

[1] Bouillaud, F., Ricquier, D., Thibault, J. and Weissenbach, J. (1985). Molecular approach to thermogenesis in brown adipose tissue: cDNA cloning of the mitochondrial uncoupling protein, *Proc. Natl. Acad. Sci. USA*, **82**, 445–448.

[2] Ricquier, D. and Bouillaud, F. (1998). Les protéines découplantes, *m/s*, **14**, 889–897.

[3] Himms-Hagen, J. (1990). Brown adipose tissue thermogenesis: interdisciplinary studies, *FASEB J.*, **4**, 2890–2898.

[4] Fleury, C., Neverova, M., Collins, S., Raimbault, S., Champigny, O., Levi-Meyrueis, C., Bouillaud, F., Seldin, M. F., Surwit, R. S., Ricquier, D. and Warden, C. H. (1997). Uncoupling protein-2: a novel gene linked to obesity and hyperinsulinemia, *Nat. Genet.*, **15**, 269–272.

[5] Gimeno, R. E., Dembski, M., Weng, X., Deng, N., Shyjan, A. W., Gimeno, C. J., Iris, F., Ellis, S. J., Woolf, E. A. and Tartaglia, L. A. (1997). Cloning and characterization of an uncoupling protein homolog. A potential modulator of human thermogenesis, *Diabetes*, **46**, 900–906.

[6] Boss, O., Samec, S., Paoloni-Giacobino, A., Rossier, C., Dulloo, A., Seydoux, J., Muzzin, P. and Giacobino, J. P. (1997). Uncoupling protein-3: a new member of the mitochondrial carrier family with tissue-specific expression, *FEBS Lett.*, **408**, 39–42.

[7] Gong, D. W., He, Y., Karas, M. and Reitman, M. (1997). Uncoupling protein-3 is a mediator of thermogenesis regulated by thyroid hormone, beta3-adrenergic agonists, and leptin, *J. Biol. Chem.*, **272**, 24129–24132.

[8] Vidal-Puig, A., Solanes, G., Grujic, D., Flier, J. S. and Lowell, B. B. (1997). UCP3: an uncoupling protein homologue expressed preferentially and abundantly in skeletal muscle and brown adipose tissue, *Biochem. Biophys. Res. Commun.*, **235**, 79–82.

[9] Mao, W., Yu, X. X., Zhong, A., Li, W., Brush, J., Sherwood, S. W., Adams, S. H. and Pan, G. (1999). UCP4, a novel brain-specific mitochondrial protein that reduces membrane potential in mammalian cells, *FEBS Lett.*, **443**, 326–330.

[10] Malaisse, W. J. (1997). In International textbook of diabetes mellitus. (Alberti, P. Z. K. G. M. M., DeFronzo, R. A. and Keen, H. Eds., John Wiley & sons Ltd.), pp. 315–335.

[11] Zhou, Y. T., Shimabukuro, M., Koyama, K., Lee, Y., Wang, M. Y., Trieu, F., Newgard, C. B. and Unger, R. H. (1997). Induction by leptin of uncoupling protein-2 and enzymes of fatty acid oxidation, *Proc. Natl. Acad. Sci. USA*, **94**, 6386–6390.

[12] Kaisaki, P. J., Woon, P. Y., Wallis, R. H., Monaco, A. P., Lathrop, M. and Gauguier, D. (1998). Localization of tub and uncoupling proteins (Ucp) 2 and 3 to a region of rat chromosome 1 linked to glucose intolerance and adiposity in the Goto-Kakizaki (GK) type 2 diabetic rat, *Mamm. Genome.*, **9**, 910–912.

[13] Surwit, R. S., Wang, S., Petro, A. E., Sanchis, D., Raimbault, S., Ricquier, D. and Collins, S. (1998). Diet-induced changes in uncoupling proteins in obesity-prone and obesity-resistant strains of mice, *Proc. Natl. Acad. Sci. USA*, **95**, 4061–4065.

[14] Glaser, B., Chiu, K. C., Anker, R., Nestorowicz, A., Landau, H., Ben-Bassat, H., Shlomai, Z., Kaiser, N., Thornton, P. S., Stanley, C. A., Spielman, R. S., Gogolin-Ewens, K., Cerasi, E., Baker, L., Rice, J., Donis-Keller, H. and Permutt, M. A. (1994). Familial hyperinsulinism maps to chromosome 11p14-15.1, 30cM centromeric to the insulin gene, *Nature Genetics*, **7**, 185–188.

[15] Bach, A., Schirardin, H., Bauer, M. and Schaeffer, A. (1981). Age-related changes in biological parameters in Zucker rats, *Lipids*, **16**, 841–848.

[16] Bernard, C., Thibault, C., Berthault, M. F., Magnan, C., Saulnier, C., Portha, B., Pralong, W., Pénicaud, L. and Ktorza, A. (1998). Pancreatic beta cell regeneration after 48 h glucose infusion in mildly diabetic rats is not correlated with functional improvement, *Diabetes*, **47**, 1058–1065.

[17] Simmons, D. M., Arriza, J. L. and Swanson, L. W. (1989). A complete protocol for *in situ* hybridization of messenger RNAs in brain and other tissues with radiolabeled single-stranded RNA probes, *J. Histotechnol.*, **12**, 169–181.

[18] Naimi, N., Rivest, S., Racotta, I. and Richard, D. (1997). Neuronal activation of the hypothalamic magnocellular system in response to oropharyngeal stimuli in the rat, *J. Neuroendocrinol.*, **9**, 329–340.

[19] Chomczynski, P. and Sacchi, N. (1987). Single step method of RNA isolation by acid guanidium thiocyanate-phenol-chloroform extraction, *Anal. Biochem.*, **162**, 156–159.

[20] Shimabukuro, M., Zhou, Y. T., Lee, Y. and Unger, R. H. (1997). Induction of uncoupling protein-2 mRNA by troglitazone in the pancreatic islets of Zucker diabetic fatty rats, *Biochem. Biophys. Res. Commun.*, **237**, 359–361.

[21] Turkenkopf, I. J., Johnson, P. R. and Greenwood, M. R. C. (1982). Development of pancreatic and plasma insulin in prenatal and suckling Zucker rats, *Am. J. Physiol.*, **242**, E220–E225.

[22] Chua, S. C., Chung, W. K., Wu-Peng, X. S., Zhang, Y., Lui, S. M., Tartaglia, L. and Leibel, R. L. (1996). Phenotypes of mouse diabetes and rat fatty due to mutation in the Ob (leptin) receptor, *Science*, **271**, 994–996.

[23] McGarry, J. D. and Dobbins, R. L. (1999). Fatty acids, lipotoxicity and insulin secretion, *Diabetologia*, **42**, 128–138.

[24] Kageyama, H., Suga, A., Kashiba, M., Oka, J., Osaka, T., Kashiwa, T., Hirano, T., Nemoto, K., Namba, Y., Ricquier, D., Giacobino, J. P. and Inoue, S. (1998). Increased uncoupling protein-2 and -3 gene expressions in skeletal muscle of STZ-induced diabetic rats, *FEBS Lett.*, **440**, 450–453.

[25] Millet, L., Vidal, H., Andreelli, F., Larrouy, D., Riou, J. P., Ricquier, D., Laville, M. and Langin, D. (1997). Increased uncoupling protein-2 and -3 mRNA expression during fasting in obese and lean humans, *J. Clin. Invest.*, **100**, 2665–2670.

[26] Barbe, P., Millet, L., Larrouy, D., Galitzky, J., Berlan, M., Louvet, J. P. and Langin, D. (1998). Uncoupling protein-2 messenger ribonucleic acid expression during very-low-calorie diet in obese premenopausal women, *J. Clin. Endocrinol. Metab.*, **83**, 2450–2453.

[27] Boss, O., Samec, S., Dulloo, A., Seydoux, J., Muzzin, P. and Giacobino, J. P. (1997). Tissue-dependent upregulation of rat uncoupling protein-2 expression in response to fasting or cold, *FEBS Lett.*, **412**, 111–114.

[28] Samec, S., Seydoux, J. and Dulloo, A. G. (1998). Role of UCP homologues in skeletal muscles and brown adipose tissue: mediators of thermogenesis or regulators of lipids as fuel substrate?, *FASEB J.*, **12**, 715–724.

[29] Wang, M. Y., Shimabukuro, M., Lee, Y., Trinh, K. Y., Chen, J. L., Newgard, C. B. and Unger, R. H. (1999). Adenovirus-mediated overexpression of uncoupling protein-2 in pancreatic islets of Zucker Diabetic rats increases oxidative activity and improves ß-cell function, *Diabetes*, **48**, 1020–1025.

[30] Johnson, J. H., Ogawa, A., Chen, L., Orci, L., Newgard, C. B., Alam, T. and Unger, R. H. (1990). Underexpression of ß-cell high Km glucose transporters in non-insulin-dependent diabetes, *Science*, **250**, 546–549.

[31] Chan, C. B., MacDonald, P. E., Saleh, M. C., Johns, D. C., Marban, E. and Wheeler, M. B. (1999). Overexpression of uncoupling protein 2 inhibits glucose-stimulated insulin secretion from rat islets, *Diabetes*, **48**, 1482–1486.

